# Information-Theoretic Concepts in Physics

**DOI:** 10.3390/e27030270

**Published:** 2025-03-05

**Authors:** Michael E. Cuffaro, Stephan Hartmann

**Affiliations:** Munich Center for Mathematical Philosophy, LMU Munich, Geschwister-Scholl-Platz 1, 80539 München, Germany; s.hartmann@lmu.de

The concepts of computation and information are becoming increasingly important, both in everyday life and in the sciences. They are at the core, for instance, of our modern understanding of the fundamentals of human and artificial cognition [1]. Social scientists have been using computational and informational concepts and techniques in their analyses of dynamic social systems with increasing frequency [2]. The same goes for physics. Computational and information-theoretic concepts and principles are, arguably, as important for understanding modern physics as the more traditional concepts and principles that have shaped the field to date [3,4]. This is true both in applied and theoretical physics. Examples include using quantum mechanical systems to perform computations and transmit information [5,6]; the use of information-theoretic concepts to characterize the structure of spacetime and gravitational phenomena [7,8]; informational axiomatisations and interpretations of quantum theory [9]; and many others.

It is with pleasure that we introduce this Special Issue of *Entropy*, entitled *Information-Theoretic Concepts in Physics*, whose aim is to provide readers with a snapshot of some of the important foundational and philosophical research at the cutting edge of this important area of physics. The seven papers included in this Special Issue cover a variety of subjects, including the nature of quantum correlations and quantum contextuality, informational and information-based approaches to interpreting quantum mechanics and its associated conceptual puzzles, causal perspectivalism, determinism, and free agency. A brief summary of each contribution can be found below.

In *Bounding Quantum Correlations: The Role of the Shannon Information in the Information Causality Principle* [10], Natasha Oughton and Christopher G. Timpson consider the information causality principle, which has been suggested as a candidate law of nature in [11]. They demonstrate that when formulated with respect to an alternative measure of information—namely, one of the Rényi measures—the principle no longer yields a correct value for the Tsirelson bound [12]. Oughton and Timpson conclude, on the basis of this and other arguments, that the information causality principle is of limited significance for foundations.

In *A New Logic, a New Information Measure, and a New Information-Based Approach to Interpreting Quantum Mechanics* [13], David Ellerman argues that the essence of the mathematics of quantum mechanics is the linearized Hilbert space version of the mathematics of partitions. In his article, Ellerman lays out the key mathematical concepts involved in the progression from logic, to logical information, to quantum theory—of distinctions versus indistinctions, definiteness versus indefiniteness, or distinguishability versus indistinguishability, which he argues run throughout the mathematics of quantum mechanics.

In *Broken Arrows: Hardy–Unruh Chains and Quantum Contextuality* [14], Michael Janas and Michel Janssen consider a family of non-maximally entangled states of pairs of particles, originally conceived of by Lucien Hardy and William G. Unruh, that give rise to correlations which cannot be accounted for in a local hidden-variable theory, and which nicely illustrate, according to Janas and Janssen, the phenomenon of quantum contextuality. Using a framework originally inspired by Jeffrey Bub and Itamar Pitowsky and developed in detail in [15], Janas and Janssen construct and analyze what they call “Hardy–Unruh chains” in terms of fictitious systems which mimic the behaviour of spin-1/2 particles.

In *Classical Information and Collapse in Wigner’s Friend Setups* [16], by Veronika Baumann, the author considers the famous “Wigner’s friend” thought experiment, whose protagonists are an observer—the friend—and a superobserver—Wigner—who treats the friend, together with her lab, as a quantum system. The so-called “Wigner’s friend paradox” points to the prima facie tension between the ways in which Wigner and his friend each describe what appear to be the identical physical circumstances, and within this context, one can prove a number of so-called “local friendliness inequalities”, similar to Bell’s theorem. In her article, Baumann shows how one can regulate the accessibility of information about Wigner’s friend’s measurements by controlling the properties of a (quasi) classical communication channel between the two of them, and how this provides a smooth transition between the various physical descriptions of the experiment.

In *The Measurement Problem Is a Feature, Not a Bug—Schematising the Observer and the Concept of an Open System on an Informational, or (Neo-)Bohrian, Approach* [17], Michael E. Cuffaro expands upon the sense in which the informational approach to interpreting the formalism of quantum mechanics, originated by Bub, Pitowsky and others, and most recently developed in [15], is (neo-)Bohrian. Cuffaro argues that on this approach, quantum mechanics represents what Bohr called a “natural generalization of the ordinary causal description”, in the sense that the idea (which philosophers of science writing in other contexts have argued for on the grounds of practical and epistemic necessity) that understanding a theory as a theory of physics requires one to “schematize the observer” within it, is elevated in quantum mechanics to the level of a postulate. After introducing and motivating this view, Cuffaro considers it, and the quantum mechanical generalization of the concept of an open system, in the light of one of Einstein’s arguments that the theory is incomplete.

In *Physical Grounds for Causal Perspectivalism* [18], Gerard J. Milburn, Sally Shrapnel and Peter W. Evans demonstrate how the asymmetry that is characteristic of causal relations can be grounded in the internal physical states of a special kind of open and irreversible physical system which they refer to as a causal agent: an autonomous physical system, maintained in a steady state, far from thermal equilibrium, with special subsystems for interacting and learning from the environment. In this context, the authors show that the learning of causal relations is driven by the thermodynamic principle that the error rate be minimized when the dissipated power is minimized, and they argue that the dependence of causal relations on such ‘hardware’ constraints constitutes a novel demonstration of causal perspectivalism.

Finally, in *Free Agency and Determinism: Is There a Sensible Definition of Computational Sourcehood?* [19], Marius Krumm and Markus P. Müller question whether free agency is compatible with determinism. It has been suggested that the principle of “computational irreducibility” hints at a positive answer, as this principle implies that, in general, there are no computational shortcuts for predicting the future behaviour of agents, which would seem to explain why deterministic agents often appear to act freely. In their paper, the authors introduce a variant of computational irreducibility which appeals to what Krumm and Müller call “computational sourcehood”—the phenomenon that the successful prediction of a process’ behaviour typically involves an almost exact representation of the relevant features of that process—and they consider what would be required to formalize the notion.
